# Pig Lung Xenotransplantation: Barriers on the Road to Clinical Translation

**DOI:** 10.3389/ti.2025.15542

**Published:** 2026-01-09

**Authors:** Sho Takemoto, Lars Burdorf, Richard N. Pierson

**Affiliations:** Center for Transplantation Sciences, Department of Surgery, Massachusetts General Hospital and Harvard Medical School, Boston, MA, United States

**Keywords:** xenotransplantation, lung, pig-to-baboon xenotransplantation, non-human primate model, decedent model

## Abstract

Lungs remain one of the most difficult solid organs for xenotransplantation, owing to its delicate alveolar capillary barrier and intense crosstalk between innate immunity and coagulation system. Multi-gene-engineered donor pig organs combined with co-stimulation pathway blockade based immunosuppressive regimen have extended xenograft survival in preclinical models using non-human primates (NHP) from hours to weeks. Most recently, the first case of lung xenotransplantation into a brain-dead human recipient was reported, confirming technical feasibility without hyperacute rejection while revealing early inflammatory injury and progressive dysfunction. Key barriers include loss of vascular barrier function, dysregulated coagulation and platelet function driven by porcine-human molecular incompatibilities, and antibody-mediated injury. Preclinical data implicate innate immune activation such as natural killer cells and macrophages. Unlike kidney xenotransplantation, which has achieved stable long-term outcomes in NHPs, lungs require attention to immunogenicity against the “fourth antigen” in triple-knockout (TKO) donors that include the positive crossmatch created by the CMAH deletion when TKO organs are tested in NHP. Although consistent multi-month lung xenograft survival has not yet been achieved in preclinical models, the remaining barriers to clinical translation are being defined. This review delineates lung-specific xeno-immune mechanisms and advances aimed at their mitigation, providing insights necessary for future clinical translation.

## Introduction

Lung transplantation is the gold standard and final therapeutic option for various types of end-stage chronic pulmonary diseases. However, the persistent global shortage of donor organs remains a critical challenge. Despite the utilization of expanded-criteria donors, including donation after circulatory death, and advancements in organ allocation systems, the waitlist mortality remains alarmingly high at over 28 deaths per 100 person-year [1]. Long waiting time and high waitlist mortality highlight the urgent need for alternative solutions. Xenotransplantation—the transplantation of organs from one species to another—has emerged as a promising alternative to address this unmet need. Recent milestones include pig‐to‐human heart [2] and kidney [3] xenotransplantation under “compassionate use” circumstances. These breakthroughs leveraged extended graft survival and function achieved in non-human primate (NHP) models using multi-genetically engineered (GE) pigs and advanced immunosuppressive regimens targeting co-stimulation pathway [4–6].

Lung xenotransplantation presents unique and formidable challenges due to the lung’s delicate alveolar architecture, large populations of resident immune cells, and inherent susceptibility to inflammation. Xeno lung differs from allo lung by triggering a host of innate immune injury mechanisms that do not normally play any significant role in allo, amplifying the deleterious consequences of inflammation and injury. Lung injury or inflammation typically leads to loss of vascular barrier function, alveolar flooding, and progressive loss of gas transfer functionality. Specifically, the immediate activation of pig lung macrophages after exposure to human blood, rapid accumulation of human neutrophils, and severe platelet sequestration and activation pose hurdles to achieving even short-term lung xenograft survival in NHP model or during *ex vivo* perfusion with human blood [7–10].

Despite multiple breakthroughs that have enabled improved preclinical results and even clinical translation of kidney and heart xenotransplantation, progress in the lung xenotransplantation has remained comparatively limited [11, 12]. Even with similar gene modifications and further intensified immunosuppressive protocols, preclinical NHP models of lung xenotransplantation (including a recent pig lung in a ‘decedent’ human) reported survival of only a few days to weeks [7, 13].

This review aims to provide a comprehensive overview of the current state of xeno-lung transplantation, highlighting recent scientific breakthroughs, ongoing challenges, and future directions. By synthesizing findings from preclinical studies and experimental models, this article seeks to inform future research and clinical translation efforts in this rapidly evolving field.

## Structural Vulnerabilities and Immunological Barriers in Lung Xenografts

The extensive surface area of lung vascular endothelium represents a primary initial target of injury in xenogeneic lung transplantation. This endothelium, intimately associated with the alveolar epithelium via a thin connective tissue layer and basement membrane, is a principal target for preformed anti-pig antibodies, “recipient” innate immune cells such as neutrophils, NK cells, and monocytes, and coagulation pathway component adhesion and activation [11]. The lungs also harbor a specialized immune surveillance network, comprised of tissue-resident macrophages, basophils, eosinophils, and other inflammatory cells that normally participate in lung tissue surveillance. While this system is highly effective in detecting and responding to pathogens, it renders the lungs uniquely susceptible to local inflammatory reactions compared with other transplanted organs. Endothelial activation and injury results in loss of vascular barrier function with alveolar flooding which prevents gas exchange in that lung region, exacerbating graft failure [14, 15]. Our prior research consistently demonstrates that inflammatory responses in both the xenograft and the recipient are significantly more pronounced for lung xenografts compared to other pig organs [5, 11, 16–27].

The intrinsic vulnerability of the lungs to xenogeneic injury arises from the interplay of innate immune pathways and interspecies physiological mismatches. Preformed antibodies, particularly those directed against the α-1,3-galactose (α-Gal) epitope, rapidly bind to donor endothelial cells, triggering complement activation and hyperacute rejection within minutes [28, 29]. This cascade leads to endothelial damage, microvascular thrombosis, and pulmonary edema, culminating in graft failure. While the genetic knockout of the galactosyltransferase enzyme (GalTKO) has effectively eliminated α-Gal-mediated hyperacute rejection in other organs [30, 31], subsequent studies revealed that innate and adaptive immune responses persist [18, 32–35]. These responses target other porcine antigens, including those derived from the N-Glycolylneuraminic acid (Neu5Gc/CMAH) and β-1,4-N-acetyl-galactosaminyltransferase 2 (β4Gal) genes [36]. Lungs from GalTKO.CMAHKO.human CD46 (hCD46) pigs, when perfused *ex vivo* with human blood, showed significant reductions in thrombin generation, thromboxane and histamine release, and pulmonary vascular resistance compared to controls without CMAHKO [37]. This genetic combination delays the onset of pulmonary vascular injury and preserves graft function [37].

Building on these findings, triple-knockout (TKO) pigs (GalTKO.CMAHKO.β4GalKO) have shown further improvements. In a notable *in vivo* experiment, lungs from these genetically engineered pigs supported baboon recipients for 5 days without the need for additional human protective transgenes [7]. In comparison, GalTKO lungs without CMAHKO or β4GalKO typically fail within 24 h and provide only marginal function [7]. While the TKO approach initially appeared to be the optimal and foundational genetic modification for xenotransplantation, subsequent research has revealed a new challenge.

Baboons and other Old World NHP possess antibodies against TKO pig cells, apparently targeting a “fourth xenoantigen” that becomes exposed following CMAHKO [38–40]. Our pilot data also support this finding: *in vivo* experiments using genetically engineered pig with 10 genetic modifications (10GE) (TKO. Growth hormone receptor knockout [GHKO].hCD46.hCD55.human endothelial protein C receptor [hEPCR].human thrombomodulin [hTBM].human heme oxygenase-1 [hHO-1].hCD47) pig lungs elicited high levels of innate immune system activation and systemic inflammation in baboon recipients (unpublished). In contrast, the longest survival observed in our *in vivo* studies (31 days) was achieved using pig lungs from donors with “double knockout” (DKO) (GalTKO.β4GalKO) [7]. These findings suggest that TKO organs—even in the widely used 10GE construct or in pigs with fewer genetic modifications—are insufficiently protected against immune-mediated injury in NHP models. However, the positive cross-matches against GalTKO + CMAHKO or TKO cells were not observed in human [39, 41].

We believe this phenomenon undermines the predictive accuracy of NHP-based transplant results for clinical outcomes, as these immune challenges are unlikely to occur in human recipients. Consequently, while NHP studies provide valuable insights, their results may underperform in reflecting the true potential of these genetically modified organs in clinical settings, emphasizing the importance of refining genetic constructs and preclinical models including work in human ‘decedents’. In August 2025, a Chinese group reported the first experimental single left lung xenotransplantation into a brain-dead ‘decedent’ human patient, observing the organ’s histologic appearance for a 9-day period. By day 1 the posterior >80% of the lung xenograft appeared to be filled with fluid, suggesting rapid loss of barrier function, similar to pig-to-baboon lungs when well-described lung xeno rejection mechanisms are not inhibited. This model, if additionally used to assess life-supporting lung function, offers the potential to yield valuable insights into the current viability of and remaining challenges for clinical lung xeno application [13].

## Inflammatory and Thrombotic Pathways in Lung Xenotransplantation

The sequestration and activation of circulating leukocytes and platelets are hallmark features of lung xenograft injury, uniquely severe compared to other xenografted organs. These processes persist even when antibody binding and complement activation are minimized, indicating that additional adhesive and activation mechanisms play significant roles in the pathogenesis [11]. Porcine endothelial cells are potent activators of human leukocytes, primarily through cytokine elaboration and species-specific incompatibilities in cellular pathways.

Interleukin-8 (IL-8), a key chemoattractant produced by porcine endothelial cells, significantly promotes neutrophil adhesion and rolling on the endothelium [42]. Elevated IL-8 levels observed in *ex vivo* pig lung perfusion models stimulate human neutrophil activation and adhesion, exacerbating pulmonary vascular resistance (PVR) and vascular barrier dysfunction [42]. Additionally, porcine endothelial selectins, such as P- and E-selectin, enhance neutrophil tethering and rolling, further amplifying leukocyte infiltration [43]. Blocking these selectin-mediated interactions with inhibitors like GM1271 (E-selectin) and rPSGL-1 (P-selectin) has shown efficacy in mitigating neutrophil-mediated damage [43].

Platelet activation plays a similarly critical role in lung xenograft injury. Porcine von Willebrand factor (pvWF), expressed and released by porcine endothelial cells, demonstrates abnormal interactions with human glycoprotein Ib (GPIb). Unlike human von Willebrand factor (hvWF), which binds weakly to GPIb under normal conditions and requires high shear stress for activation, pvWF activates quiescent human platelets even under low shear stress. This aberrant interaction leads to pathological platelet aggregation and microvascular thrombosis, significantly contributing to the prothrombotic environment within the xenograft. Such conditions exacerbate vascular occlusion and graft injury, creating a formidable barrier to long-term lung xenograft survival [44].

Donor lung pretreatment with desmopressin (DDAVP), as reported by a Korean group, reduces platelet activation by depleting pvWF from endothelial cells and minimizing its interaction with GPIb [45]. Our *ex vivo* lung perfusion studies further support this approach, demonstrating that pre-depletion of pvWF using DDAVP, combined with the administration of GPIb antagonists, effectively attenuates platelet activation [20]. Moreover, humanizing pvWF by replacing a portion of the gene region encoding the GPIb-binding site with its human analogue in GalTKO.hCD46 pig lungs has been shown to suppress non-physiological human platelet aggregation and sequestration within the pig lung and liver [46]. This genetic modification represents a pivotal advancement, providing a safer and more effective approach to lung xenotransplantation. Importantly, it may also facilitate xenotransplantation applications for other organs and cells, broadening its potential clinical impact.

## Coagulation Cascade Activation and Dysfunctional Thromboregulatory Mechanisms

Coagulation abnormalities are a major contributor to lung xenograft injury, driven by endothelial cell activation or damage, which triggers the coagulation cascade and leads to rapid thrombus formation. Key factors implicated in this process include TBM, EPCR, and TFPI. Although porcine TBM can bind human thrombin to form a thrombomodulin-thrombin complex, its protein C activation efficiency is only 1%–10% that of hTBM [47, 48]. Similarly, porcine EPCR and TFPI, which inhibit extrinsic coagulation pathway factors, exhibit significantly reduced activity compared to their human counterparts [47, 49–52].

Our *ex vivo* perfusion model using human blood demonstrated that expressing hTFPI in GalTKO pig lungs effectively suppressed neutrophil activation and provided protective effects, supporting the hypothesis that human-derived coagulation regulatory factors are critical for mitigating graft injury [10]. Baboon *in vivo* models further revealed that co-expression of hEPCR and hTBM was associated with reduced β-thromboglobulin (βTG) levels, consistent lung survival beyond 12 h, and a higher rate of achieving initial life-supporting xenograft function [7]. However, these advances can only delay the onset of barrier dysfunction and fail to completely prevent it.

Early production of thromboxane and histamine also contributes to loss of barrier function [19]. Treatment with the selective thromboxane inhibitor 1-benzylimidazole (1-BIA) combined with H-2 or non-selective histamine receptor antagonists significantly suppressed PVR elevation and delayed vascular barrier dysfunction [21]. However, the combination of drug regimens targeting inflammatory cytokines such as tumor necrosis factor-α (TNF-α), IL-8, and IL-6 have not fully eliminated these mediators, and barrier dysfunction persists. These findings highlight the need for further investigation into the mechanisms driving coagulation abnormalities and inflammation to develop more effective therapeutic strategies.

## Complement-Mediated Lung Xenograft Injury

Complement activation is particularly pronounced in lung xenotransplantation due to the organ’s high vascular density and unique susceptibility to immune-mediated injury. To prevent some protection against activation of the complement pathway, human complement regulatory proteins (CPRPs)—including decay accelerating factor (DAF_CD55), membrane cofactor protein (MCP_CD46), and membrane-attack-complex-inhibitory protein (MAC-IP_CD59)—have been introduced into the organ source pigs [53]. (Porcine CPRPs are not very effective at controlling human complement activation due to interspecies molecular incompatibilities and differences in their expression levels on vascular endothelium [54, 55]).

In addition, several pharmacological approaches have been explored to mitigate complement activation in lung xenografts, including the use of C1-esterase inhibitor, soluble complement receptor 1 (sCR1), FUT-175, and depleting agents such as cobra venom factor (CoVF). While these strategies have demonstrated partial success, none has provided a definitive solution [56–58]. To address this challenge, genetic introduction of human complement regulatory proteins (hCPRPs), such as CD46, CD55, and CD59, has been investigated. When combined with DKO or TKO backgrounds, these genetic modifications have shown promising results in preclinical *in vivo* models. Specifically, hCPRPs expression in xenografts has been associated with reduced complement deposition, decreased platelet activation, and delayed graft injury in lungs, as well as in other organs such as the heart and kidneys [19, 59, 60].

## Self-Recognition and Xenograft Injury by Macrophage and Natural Killer Cell

Cellular immune mechanisms are central to lung xenograft injury, involving macrophages and natural killer (NK) cells. Signal regulatory protein alpha (SIRPα), a key inhibitory receptor expressed on macrophages, plays a crucial role in distinguishing self from non-self. Interaction between SIRPα and its ligand, CD47 prevents autologous phagocytosis [61, 62]. However, in the absence of this interaction, porcine cells become highly susceptible to phagocytosis by human macrophages. Introducing human CD47 into porcine cells significantly reduces this susceptibility and effectively inhibits macrophage-mediated phagocytosis [63, 64]. Yamada et al. further reported that expressing CD47 in porcine lungs extended chimerism after bone marrow transplant and improved xeno-lung recipient survival in baboons [65]. While Watanabe et al. reported up to 10-day survival in baboon recipients using a GalTKO base with hCD47 expression alone, our series using GalTKO.hCD46 backgrounds with hCD47 alone or in combination with other humanized proteins (e.g., hEPCR, hTBM, hCD55, human tissue factor pathway inhibitor [hTFPI], and HO-1) failed to achieve consistent survival beyond 2 days [7]. Anatomical localization of hCD47 within the graft may influence its efficacy, warranting further investigation [66].

Pre-harvest donor macrophage depletion using liposomal clodronate has shown attenuation of acute ischemia reperfusion injury in a mouse lung allotransplant model and prevented endotoxin-induced acute lung injury in pigs showing significantly lower levels of TNF-α, IL-6, and thrombin [67, 68]. When it is applied to xenotransplantation, macrophage depletion significantly attenuates hyperacute rejection in wild-type pigs [22, 69]. In our *in vivo* baboon model, not only the use of liposomal clodronate but also anti-pig antibody absorption and cytokine inhibition were associated with longer xeno-lung recipient survival [7].

In addition to CD47, human CD39 and CD73 have emerged as promising anti-inflammatory mediators. These molecules convert extracellular pro-inflammatory ATP into AMP, reducing inflammation and vascular constriction [70, 71]. Genetically engineering porcine lungs to express human CD39 and CD73 could further suppress inflammation and enhance graft survival by modulating the graft’s immune microenvironment.

Natural killer (NK) cells play a dual role in xenograft injury by identifying and lysing non-self cells through both antibody-dependent and independent mechanisms. A critical factor driving NK cell activation in xenografts is the incompatibility between human inhibitory receptors and the porcine major histocompatibility complex (MHC), also known as swine leukocyte antigens (SLA). This weak interaction fails to deliver the necessary inhibitory signals, leaving porcine cells vulnerable to NK cell-mediated destruction [72]. Moreover, the absence of human leukocyte antigen E (HLA-E) on porcine endothelial cells exacerbates NK cell activation by preventing the recognition of negative regulatory signals [73, 74]. In contrast, lungs from GalTKO.hCD46 pigs expressing HLA-E demonstrated substantial protection against NK cell attacks both *in vitro* and in an *in vivo* model, leading to reduced early graft injury and prolonged survival in preclinical models [74–76].

## Immunosuppressive Regimen and Targeted Drug Therapies

Optimal immunosuppressive regimens for lung xenotransplantation remain undefined. Building on the promising outcomes of co-stimulation pathway blockade in cardiac and renal allo- and xenotransplantation [2, 5, 77–81], strategies targeting CD154/CD40 and CD28/B7 pathways have been investigated *in vivo* lung xenograft models [7]. While these approaches have shown potential in modulating adaptive anti-xeno immunity, they are insufficient as standalone therapies. Consequently, co-stimulation blockade has been combined with conventional immunosuppressive therapies commonly used in human organ transplantation, including pre-transplant induction with antithymocyte globulin (ATG) and anti-CD20 (rituximab), along with maintenance regimens comprising steroids and mycophenolate mofetil (MMF). Additionally, splenectomy is often performed in our *in vivo* models to reduce the reservoir of recipient B-cells available to generate *de novo* anti-pig antibodies as well as spleen-resident plasma cells responsible for elaboration of preformed ‘innate’ anti-pig antibodies, thus hoping to enhance immunosuppressive regimen efficacy [7].

To address the unique inflammatory mediators that we have observed to be elaborated in association with lung xenografts, various anti-inflammatory agents have been explored. We now consistently include anti-TNF-alpha (etanercept), anti-IL-8 (reparixin), anti-IL-6 receptor (tocilizumab), C1-esterase inhibitors, and alpha1-proteinase inhibitors based on the profile of cytokines we have measured in earlier work. Of note, consistent incorporation of all 4 of these reagents is not by itself sufficient to achieve consistent improvement in xenograft survival or prevention of initial barrier dysfunction [7].

A significant milestone in the field was reported in August 2025, when researchers in China conducted the first lung xenotransplantation in a brain-dead human recipient using a 6GE pig lung (TKO.CD55.CD46.TBM) [13]. The immunosuppressive regimen included induction with ATG and a tacrolimus-based protocol, supplemented with MMF, steroids, anti-IL-2 receptor (basiliximab), anti-C5 (eculizumab), Janus kinase (JAK) inhibitor (tofacitinib), and anti-CD80/86 (belatacept). Within the first 24 h radiologic imaging revealed consolidation of the majority of the lung xenograft. We suspect that resident pig lung macrophage activation triggered histamine and thromboxane elaboration, contributing to significant edema and alveolar damage. In addition, neutrophils, NK cells, and monocytes likely infiltrated the lungs and caused additional inflammation. While deposition of immunoglobulins suggesting antibody-mediated rejection (AMR) was not clearly observed until day 3 we suspect antibody deposition and complement activation were likely present earlier. The authors suggest that there were signs of improvement in parenchymal damage by day 9, but evidence to support this interpretation we do not find compelling. Although this is a single case report, it highlights two critical priorities for advancing lung xenotransplantation: it is essential to measure lung function in addition to histology in order to accurately predict likely performance of a pig lung xenograft implanted with therapeutic intent; and controlling severe inflammation, including AMR, during the first week post-transplant was not accomplished by the regimen this team tested.

In contrast to lungs, *in vivo* baboon models of cardiac and renal xenotransplantation have demonstrated prolonged graft survival with less intensive immunosuppressive and anti-inflammatory regimens [5, 76–81]. Elucidating the unique vulnerability of lung xenografts to inflammation and rejection by tailoring specific therapies to address them will be pivotal in advancing lung xenotransplantation toward clinical application. In Table 1 we summarize our view of the remaining major barriers, the strategies we have tested to date, and next steps as we have prioritized them, which we hope will allow us to accomplish consistent long-term lung xenograft survival in our preclinical model and, eventually, in humans.

**TABLE 1:** Known barriers to durable lung xenograft function, mitigation strategies tested to date, and planned next steps.

Key issue	Key issue	Mitigation strategies deployed to date (genetic/pharmacologic)	Address gaps with next steps
Loss of vascular endothelial barrier function reflects the integrated consequence of these processes	Adaptive immune responses		
	Elicited cellular and humoral immunity against pig antigens	Safe, effective immunosuppressive regimen based on co-stimulation-blockade-based regimen	Verify anti-CD154 levels, test higher doses and additional drug combinations so as to prevent elicited anti-donor antibodies and optimize maintenance immunosuppression
	Innate immune responses		
	Dysregulated coagulation	hTBM/hEPCR/TFPI Thromboxane synthesis inhibitor	Optimize endothelial gene expression levels, evaluate different gene combinations alone and with goal-directed anticoagulation therapy
	Platelet activation and sequestration	Donor vWF depletion with desmopressin Humanized vWF; GPIb blockade	Incorporate humanized vWF into existing multi-GE pig lines Optimize anti-GPIb fab dosing
	Complement activation	hCD46, hCD55, hCD59 and other human transgenes; complement depletion with cobra venom factor transitioning to C1 esterase inhibitor, C3 or C5 inhibitors	Optimize pharmacologic drug dosing algorithms, evaluate C3 and C4 inhibitors Evaluate different combinations of human complement regulatory transgenes at optimized expression levels in donor pig lungs
	Injury mediated by preformed antibody	Carbohydrate xenoantigen deletion: α-Gal/β4GalNT2/CMAH Adsorption of preformed antibody by donor kidney perfusion B-cell depletion with rituximab; splenectomy	Standardize crossmatch gating and non-Gal antibody profiling Evaluate CMAH-intact double-knockout (GalTKO.β4GalKO) lungs with 6 or more human transgenes targeting complement, coagulation, inflammation Plasma cell depletion or inhibition
	Cytokine elaboration	Blockade of IL-6R, IL-8R, TNF-α α1 -proteinase inhibitor	Test IL-1R, IL-33 antagonists as add-on to inhibit cytokine elaboration or block effects Explore cytokine absorption using blood filters
	NK cells	HLA-E transgene to engage human CD94/NKG2A inhibitory signaling	Verify NK functional readouts (degranulation, cytotoxicity) in EVLP/NHP, and molecular evaluation of lung xenografts Evaluate CD38 depletion
	Pulmonary macrophages	hCD47; donor macrophage depletion with liposomal clodronate	Develop strategies to inhibit recipient monocyte/macrophage influx, activation Add human-compatible SIRPα, hCD73 to pig edits
	Neutrophils	Blockade of P/E-selectin/PSGL-1 and Mac-1 (CD11b/CD18)	Validate drug engagement, efficacy to inhibit targeted pathway; develop strategies to inhibit NETs Explore adding hCD200 to pig edits

Abbreviations: β4GalNT2: β-1,4-N-acetyl-galactosaminyltransferase-2; CMAH: cytidine monophosphate-N-acetylneuraminic acid hydroxylase (Neu5Gc); EVLP: *ex vivo* lung perfusion; GPIb: glycoprotein Ib; hCD46/hCD47/hCD200: human cluster of differentiation 46, 47, 200; hEPCR: human endothelial protein C receptor; hTBM: human thrombomodulin transgenes; HLA-E: human leukocyte antigen-E; IL: interleukin; KO: knockout; Mac-1: integrin αMβ2; NETs: neutrophil extracellular traps; NK: natural killer; NHP: non-human primate; PSGL-1: P-selectin glycoprotein ligand-1; SIRPα: signal regulatory protein-α; TNF- α: tumor necrosis factor- α; TFPI: tissue factor pathway inhibitor; vWF: von Willebrand factor.

## Conclusion

Multi-gene engineering of donor pig and mechanism-based adjuncts have extended experimental lung xenograft survival from hours to days and, in selected NHP models, to weeks of recipient survival. The first pig-to-human lung xenotransplantation in a brain-dead recipient confirmed technical feasibility without hyperacute rejection but underscored persistent lung-specific barriers, notably early vascular-barrier failure, coagulation and platelet dysregulation, and antibody-mediated injury. Evidence from preclinical studies also indicates roles for NK cells and for macrophages in acute injury pathways. Progress toward clinical exploration will need further improvement of outcomes in preclinical models. In addition to evaluation of TKO-based multi-GE pig lungs in decedents, we anticipate that results with DKO (GalTKO.b4GalKO.CMAH-intact) multi-GE pig genotypes in NHP models will better predict clinical performance of lung (and other organ) xenografts. Working in parallel in NHP and decedent models, we hope to facilitate identification of gene and drug combinations that are necessary and sufficient to effectively address and dependably overcome species-specific lung xeno barriers.
